# The structure of classes in Uruguay, 1963–1996: a structural–historical perspective

**DOI:** 10.3389/fsoc.2026.1787966

**Published:** 2026-08-18

**Authors:** Marcelo Boado, Sofía Vanoli, Rafael Rey

**Affiliations:** Universidad de la República, Montevideo, Uruguay

**Keywords:** class analysis, educational attainment, social inequality, structural social mobility, Uruguay censuses

## Abstract

This paper focuses on selected analytical aspects of social mobility from a structural–historical perspective, which provide important inputs for our broader research on intergenerational individual social mobility in Uruguay. We present the results of a structural–historical analysis of social classes in Uruguay during the second half of the twentieth century, based on census data on the Economically Active Population (EAP) from 1963, 1975, 1985, and 1996. Social classes are classified using Torrado’s scheme and harmonized with the structural perspective proposed by Filgueira and Geneletti (FG). While acknowledging the contributions of Terra, Errandonea, and Bazzi, this study examines the period 1963–1996 in greater depth, assessing the implications of several hypotheses previously formulated by FG. The paper builds on earlier methodological work, which details the harmonization of census microdata, the construction of a unified census database, and the development of a standardized occupational dictionary. Given the broad temporal scope of the analysis, which allows for the assessment of long-term structural hypotheses, we focus on static intertemporal contrasts for reasons of analytical clarity and conceptual consistency. Our findings support the following conclusions: (1) the central role of structural transformations and their sectoral effects; (2) the increasing relevance of the service sector—amid agricultural and industrial stagnation—for opportunities for structural social mobility; (3) the growing mismatch between educational attainment and occupational positions; (4) the massive and dual incorporation of women into the EAP; (5) the substantial decline of wage employment and rural occupations; and (6) the unequal use of educational opportunities across social classes, gender, and selected birth cohorts.

## 1 Economic development and social classes

Since World War II (WWII), the world has undergone profound political, economic, and communicational transformations. The debate between capitalism and communism was not merely an ethical dispute over political organization, but rather a confrontation over the quality and quantity of life that governments could offer their populations within increasingly participatory socio-political systems. The reconstruction of Europe, the end of colonialism, and the emergence of a new international economic and institutional order reshaped global development trajectories.

Development became both a central value and a primary objective for governments across different societies, and it was actively promoted by institutions such as ECLA (CEPAL), ILPES, and FLACSO, as well as by numerous organizations and universities. These institutions supported scholars concerned with Latin America’s position and role in the post-war economic order and with the structural roots of its social problems. As a result, the role of the State, economic growth, and class inequalities came to define the “development agenda,” along with policies aimed at reducing inequality through education, health, fertility, housing, and labor market regulation.

Within this framework, education was increasingly emphasized in both politics and the social sciences as the principal equalizer of opportunities and the preferred solution to income distribution problems, rather than land reform or changes in the ownership of productive assets. Consequently, equality of educational opportunity became a feasible policy objective adopted by most governments, in a manner unprecedented in human history. Social mobility and the possibility of improving one’s life chances thus became central goals of a development path based on global economic and commercial growth, explicitly distancing itself from communist alternatives.

Social mobility is currently studied through three main approaches: a structural–historical perspective, an intergenerational retrospective individual perspective, and a longitudinal or cohort-based perspective. This paper focuses on the first approach, which examines intertemporal changes in the distribution of the population across social classes, or changes in class size. The second approach, not addressed here, relates individuals’ occupational achievements to their social, familial, and work backgrounds over the life course. The third approach, also beyond the scope of this study, follows cohorts of individuals of the same age over time, analyzing their economic and social trajectories and the impact of macro-social events.

Drawing on the work of Filgueira and Geneletti (1981), Terra (1983), Errandonea (1989), and Bazzi (2017), this paper presents the results of a structural–historical analysis aimed at constructing a reliable measure of the evolution of Uruguay’s class structure. The analysis is based on EAP members recorded in the population censuses of 1963, 1975, 1985, and 1996. This long-term comparative exercise allows us to examine economic, social, and political changes in light of the theoretical conclusions advanced by the aforementioned authors.

Terra conducted an influential study of social classes and income distribution using data from the 1963 census and its coverage survey. Like the present study, Terra employed a broad class scheme, incorporating dimensions beyond occupation, such as power, education, income, and cultural assets. Errandonea compared the evolution of class structure using the 1975 and 1985 censuses, focusing on power relations and economic inequality among classes; his analysis was primarily descriptive and did not advance a general theoretical position.

The proposal advanced by Filgueira and Geneletti (hereafter FG) was more comprehensive and analytically systematic, and therefore particularly useful for the present study. FG argued that Latin America experienced economic growth characterized by advances in urbanization, industrialization, and the expansion of services and education, alongside the persistence of inequalities that constrained equal access to the social opportunities typically associated with growth, such as income. Their analysis covered several countries, relying on data that were far more limited than those currently available.

Rejecting the linear assumptions of modernization theory—which posited a gradual and cumulative process of socio-economic improvement—FG focused instead on the “asynchronicities” inherent in development processes. They moved beyond the strong economic determinism that viewed industrialization as the sole path to development and well-being. In particular, they argued that industrialization in Latin America was not capable of indefinitely absorbing rural–urban migration. Instead, the expansion of the tertiary sector and the growing role of the State in employment and economic activity pointed to alternative paths for overcoming “dynamic insufficiency” (resulting from unequal exchange) and for understanding long-term modernization.

Beyond the undeniable process of deruralization, and despite the implementation of agrarian reforms of varying scope across countries, the urbanization of the EAP led to a profound reorganization of occupational structures. This included the expansion of services, administration, and skilled professions; the relative stability of traditional industry; and increased state intervention in key industries and services. In this sense, FG challenged industrialist theories of development, arguing that the sequential transition from agriculture to manufacturing would not necessarily generate economies of scale—a claim that has since been widely questioned—and that alternative development paths could emerge in parallel or even more rapidly.

From this perspective, social stratification is not merely a set of labor categories but a relational system in which social positions are constructed and reproduced within dynamic and unequal production processes. FG’s attention to the middle and upper classes—often viewed as prototypes of development—highlighted their uneven distribution across economic sectors and the differing speeds of their expansion across countries. Their framework offers important insights into structural mobility: while it helps explain the decline of agricultural employment, the growth of services, the rise of professional occupations, and the feminization of the EAP, it also reveals key contradictions related to industrialization, education, and public employment.

According to FG, industrialization does not lead to unlimited growth of the middle classes or to unbounded social mobility, as these processes are constrained by broader dynamics of global growth and urbanization. Occupational differentiation is limited not only by labor supply factors such as fertility and migration, but also by structural ceilings. Drawing on earlier work by Germani (1963), Di Tella (1962), and Heintz (1970), FG warned that educational expansion tends to outpace labor market demand, generating status inconsistencies between education, occupation, and income, with potentially destabilizing political consequences. Similarly, they argued that the expansion of public employment—once viewed negatively but increasingly central in Latin America after WWII—was constrained by the nature of the dominant state and its political regime.

Despite the limitations of their stratification scheme, FG’s general framework highlights enduring tensions and contradictions in the structural–historical analysis of social mobility. Although we adopt a different class scheme, discussed later in the paper, their contribution prompts a systematic re-evaluation of our previous findings (Boado and Vanoli, 2024) and informs the following hypotheses:

1. Social mobility is induced by macroeconomic transformations, which are not always led by the secondary sector of the economy.
2. The middle class exhibits significant internal segmentation, with a propensity toward occupational status inconsistency in certain segments relative to education and income levels.
3. The growth of the middle classes slows over time, producing an “inverted J” pattern.
4. Educational expansion and occupational structure develop at different and independent rhythms, enabling unequal use of educational credentials.
5. Growth of the EAP is accompanied by persistent gender inequalities.

## 2 Macro social and historical trends in Uruguay at the end of the twentieth century

In order to assess whether the structural–historical hypotheses regarding Uruguay’s class structure between 1963 and 1996 can be verified or supported, it is necessary to outline the main macroeconomic and macrosocial variables that characterized the country’s performance during this period and to reflect on their consequences for class dynamics. To this end, we draw on multiple data sources, guided by the methodological decision to situate census-based observations within a broader temporal framework that captures the cohort experiences underlying each census.

The figures presented in this section synthesize estimates derived from various sources, including the Maddison Project (2014), Moxlad (2017), Barro and Lee (2010), and Errandonea (2014). Together, these sources provide relevant indicators for assessing economic development, modernization, structural change, and the outcomes anticipated by earlier theoretical forecasts. Whenever possible, Uruguay’s indicators are compared with regional benchmarks, either through country-level data (Argentina) or aggregated measures for the rest of South America.

At the level of Latin American economic doctrine, it is relatively straightforward to identify a broad sequence of historical phases: the period of Unequal Exchange, followed by Import Substitution Industrialization (ISI), then Dynamic Insufficiency, and finally Economic Openness and the formation of regional trade blocs. However, within Uruguayan social science, agreement on such a precise periodization is more elusive, except in very general terms. This difficulty stems from limitations in available data and discrepancies between national statistics and OECD sources.

The EAP data provided by the censuses of 1963, 1975, 1985, and 1996 correspond to a period of profound transformation in Uruguay, marked by the implementation of government policies that were often politically contested. For analytical purposes, at least two broad sub-periods can be identified. The years 1963 and 1975 reflect the final phase of the ISI model, which coincided with a period of severe social, political, and economic crisis triggered by the collapse of Uruguay’s primary and weakly diversified export base. This crisis unfolded at a time when the international economy was reorganizing its leading sectors—industrial production, food and energy systems, technology, and communications. As noted by Errandonea (1989), this process began prior to 1963 and culminated in a prolonged dictatorship during the 1970s and early 1980s, alongside a reconfiguration of Uruguay’s position in the international economy.

After 1975, and largely outside the major trade blocs emerging in Europe, Uruguay’s protected national industrial economy gradually eroded. The country opened its economy to an unstable international market, largely dependent on food exports and lacking domestic energy sources. During the 1980s and 1990s, a second period unfolded, characterized by deeper economic liberalization and Uruguay’s integration into Mercosur, within a highly asymmetric regional context. Over this period, a substantial process of deindustrialization was completed, leaving only export-oriented value chains linked to primary commodities. The censuses of 1985 and 1996 capture this transition. Uruguay remained in this position until the emergence of the BRICS economies in the early twenty-first century. Although this later phase is beyond the scope of the present analysis, it is worth noting that the expansion of global food demand led to a renewed export-driven agricultural boom, generating significant economic growth without reinstating the industrializing and protectionist model that had prevailed during the first half of the twentieth century.

**Figure 1:**
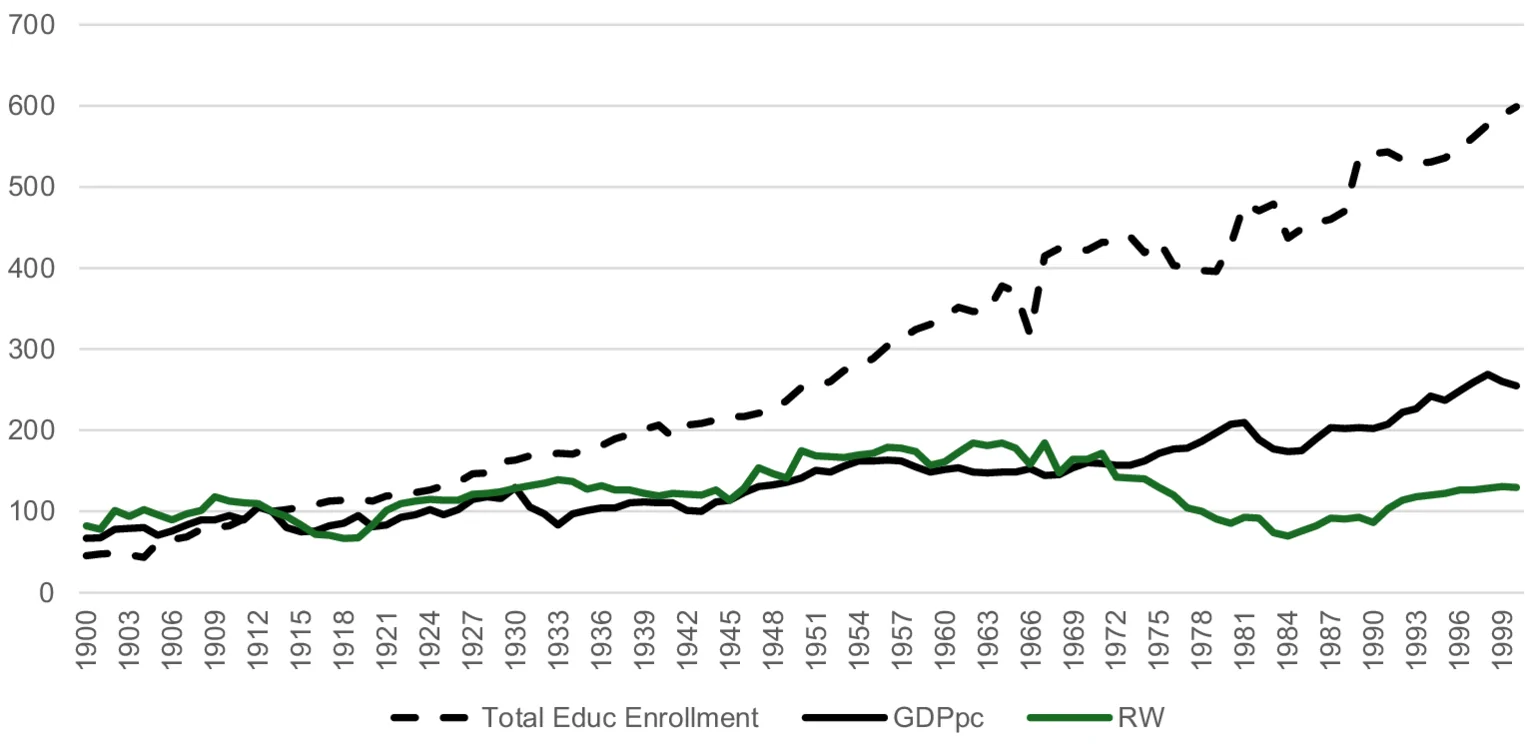
Uruguay 1900-2008 growth index rates of total educational enrollment (Prim+Sec+Tert+Univ), GDP per capita (GDPpc) and Real Wage (RW). Base 1913 = 100. Source: Authors’ elaboration based on Moxlad, Maddison Project, and Errandonea (2014).

**Figure 2:**
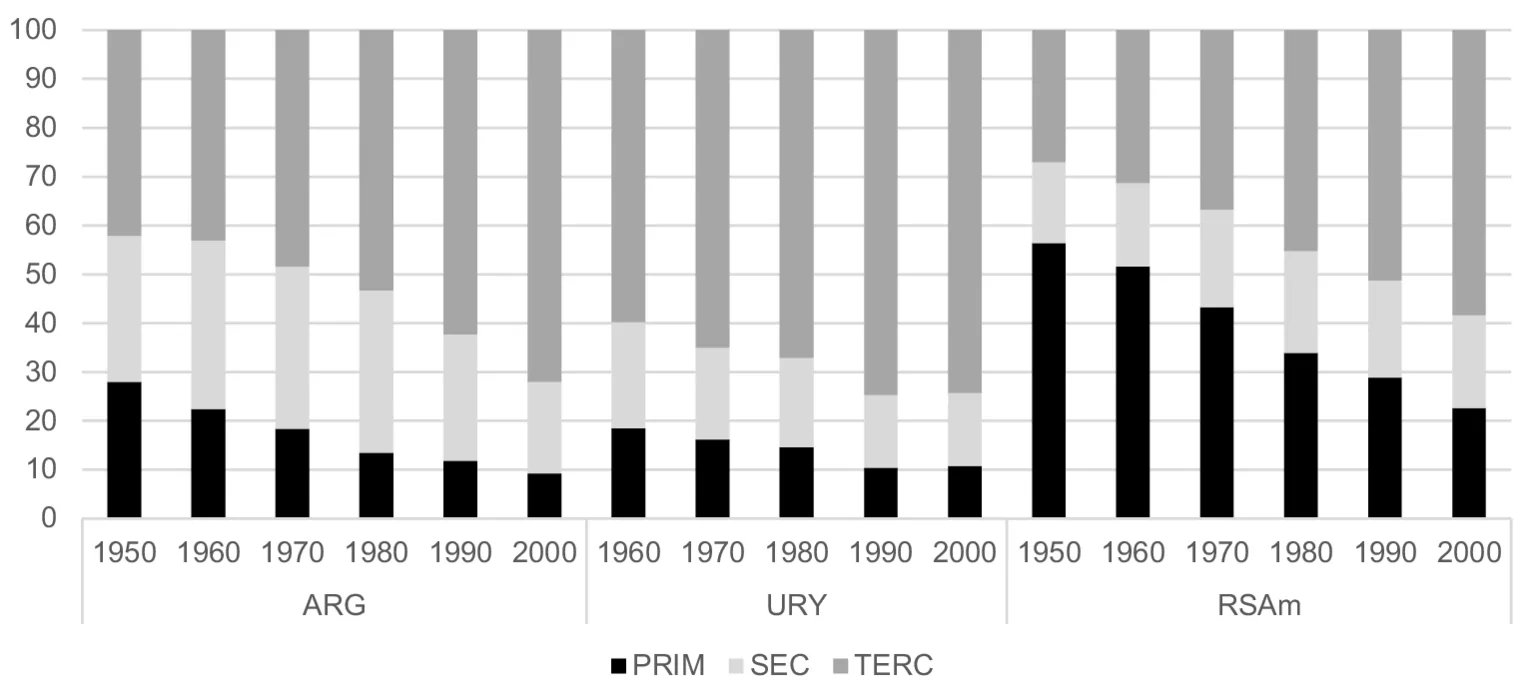
Employed EAP by big economic activity sectors 1950-2000, for Argentina, Uruguay and rest of South America. Source: Bolt and van Zanden (2014) & INE (Uruguay).

Figures 1, 2, together with Table 1, summarize the evolution of key macroeconomic variables across these stages and illustrate their implications for the social positions of multiple generations. These indicators are based on the aforementioned sources of macroeconomic estimates.

**Table 1:** Total EAP by size, years and sex.

Census years				
EAP	1963	1975	1985	1996
Total	984.461	1.077.468	1.143.470	1.414.005
Women	242.450	303.104	376.508	573.193
Men	742.011	774.364	766.962	840.812

Source: Own elaboration based on INE.

Figure 1 presents growth rates of GDP per capita (GDPpc), real wages^1^, and total educational enrollment for Uruguay. Throughout the twentieth century, GDP growth was slow and closely tracked real wages until the 1960s, after which the two series diverged permanently. Productivity and savings capacity did not increase substantially, as occurred in other regions, but neither did they collapse, instead converging to some extent with Argentina and Chile (Barozet et al., 2021). The downside of this trajectory during the 1960–1980 period—the final stage of ISI—was a sustained decline in real wages, which only began to recover in the early 1990s. This pattern reflects a significant transfer of income from labor to capital at the aggregate level, driven by the economic crisis and political tensions of the period (Errandonea, 1989; Marmisolle et al., 2023).

Gross educational enrollment reflects the proportion of the population participating annually in the education system with the expectation of improving future well-being. More broadly, it illustrates a general policy orientation in Uruguay—and in many independent countries—toward promoting productivity through institutional mechanisms that regulate factor remuneration without coercion. The data reveal a sustained upward trend in educational expansion, with fluctuations broadly aligned with GDP dynamics. However, FG’s forecast is also confirmed: educational expansion can proceed independently of productive growth, potentially generating structural bottlenecks. If Dynamic Insufficiency represents one dimension of development constraints, educational expansion represents another, and their interaction becomes evident in the sectoral evolution of the employed EAP.

Figure 2 shows changes in Uruguay’s EAP across three major economic sectors between 1950 and 2000, compared with Argentina and the rest of South America, using Maddison Project GGDC and INE (Uruguay) data. At the beginning of the period, deruralization was already more advanced in Argentina and Uruguay than in the rest of the region, and this process continued until reaching relative stabilization. In both Argentina and Uruguay, the secondary sector developed earlier and under greater protection than in the rest of South America. However, from the 1980s onward, Uruguay experienced accelerated deindustrialization following economic liberalization, while industrial employment continued to expand elsewhere in the region.

A distinctive feature of Uruguay is the size of its tertiary sector, which accounted for approximately 60% of employment as early as 1960—a level Argentina would only reach in the 1990s. Employment growth in Uruguay has been driven primarily by this sector. Its long-standing importance is supported both by early assessments (Solari, 1964) and by more recent reconstructions of GDP by sector (Bonino et al., 2012). Annex Figure A1 illustrates the evolution of average years of schooling for individuals aged 15 to 64 in Uruguay, Argentina, and the rest of South America, based on the Barro and Lee database.

Taken together, these trends point to several distinctive features. The expansion of the tertiary sector was not accompanied by a proportional increase in highly specialized occupations, as anticipated by FG. Instead, human capital formation was concentrated at basic and intermediate levels and did not translate into the productivity gains predicted by modernization theory. Although access to all levels of education expanded—supported by Uruguay’s free public education system—this expansion did not necessarily translate into improved social mobility opportunities. As shown in Annex 1, average educational attainment rose steadily from the 1950s onward but exhibited a slowdown toward the end of the twentieth century, a pattern also observed in years-of-schooling measures (Boado, 2009; Boado and Fernández, 2010; Boado and Vanoli, 2024).

Fluctuations driven by labor market dynamics further support FG’s nuanced view of development. In 1963, individuals with 13 or more years of schooling accounted for less than 4% of the EAP; by 1996, this figure had increased to 14%. This expansion indicates that educational attainment did not yield the returns predicted by modernization theory, neither in terms of meritocratic mobility nor in terms of equality of opportunity.

Table 1 shows the magnitude of intercensal growth in Uruguay’s total Economically Active Population (EAP) and highlights two key features: its overall expansion and the incorporation of women into the labor force, based on INE data. Across the four censuses, the EAP increased steadily, from just under one million individuals in 1963 to nearly one and a half million in 1996. It should be noted that while the total population grew at an average annual rate of 0.54%, the EAP expanded at a significantly higher rate of 1.1% per year.

The gender composition of the EAP changed substantially over the period, and a large share of its overall growth was driven by the increasing participation of women. Women accounted for 25% of the EAP in 1963, rising to 40% by 1996, indicating a profound transformation in labor market participation patterns.

## 3 Measuring social classes in Uruguay at the end of the twentieth century

The aforementioned studies by Terra, Filgueira and Geneletti (FG), Errandonea, and Bazzi propose different class schemes^2^, a situation that is common in the field and has been discussed elsewhere (Boado and Vanoli, 2024).

To measure social classes over the long term, we followed several analytical steps. First, we harmonized data from the four censuses into a single dataset and constructed a unified occupational dictionary^3^ applicable to all of them. The resulting database brings together more than 4.5 million EAP records from across the country, for both sexes, aged between 20 and 70 years. This structure allows for consistent analyses by cohort, educational attainment, branch of economic activity, and geographic and temporal variables, using a common occupational coding system based on two-digit ISCO-88 classifications.

The second step involved applying a reliable, flexible, and widely recognized social class scheme. For pragmatic reasons, we follow Bazzi (2017), who adapted Torrado’s (1992) Socio-Occupational Strata scheme at the individual level rather than at the household level or based on household heads or income recipients. The classification relies on a tabulation that combines occupation, occupational category, branch of activity, and size of productive unit (Torrado, 1992; Sacco, 2019), resulting in eight Socio-Occupational Strata (hereafter ESO).

We introduced additional refinements that expanded the original eight-category ESO scheme to twelve categories, allowing for greater differentiation in terms of wage levels and sectoral characteristics across the four censuses. The detailed procedures are documented in Boado and Vanoli (2024), and Annex Table A2 presents the operational tabulator applied in this study. Although the ESO scheme differs from that used by FG, we reorganized the ESO categories to approximate their major class divisions, facilitating the empirical assessment of FG’s hypotheses for Uruguay over a longer historical period than originally examined.

The third step consisted of analyzing intertemporal contrasts among the ESO categories according to elapsed time, sex, and the educational attainment of selected cohorts. These analyses are presented in the following sections.

The twelve ESO categories generated are described below, and their absolute and relative distributions are reported in Table 2. The white and grey internal lines in the Table 2 indicate the reorganization of ESO categories to approximate the broader class groupings used by FG.

**Table 2:** Absolute and relative distributions of ESO of both according to censuses, with categories roughly reordered to Filguiera and Geneletti.

ESO (re ordered for FG)	1963		1975		1985		1996	
	Frec	%	Frec	%	Frec	%	Frec	%
Company directors, manager, high officials	4,866	0,5	6,636	0,7	5,318	0,5	4,882	0,4
Professionals in specific function	13,265	1,4	19,577	2,0	30,515	2,8	35,420	3,1
Small business owners	45,295	4,9	49,796	5,0	48,827	4,5	47,148	4,2
Technical and similar cadres	99,556	10,7	89,933	9,1	129,249	12,0	129,577	11,5
Armed forces	13,142	1,4	29,826	3,0	29,916	2,8	21,062	1,9
Administrative employees and salespeople	100,436	10,8	127,732	12,9	134,256	12,4	143,481	12,7
Agricultural producers	69,232	7,4	68,783	6,9	72,107	6,7	61,411	5,4
Self-employed workers	90,146	9,7	95,490	9,6	102,470	9,5	142,527	12,6
Skilled workers	228,930	24,6	226,302	22,8	264,066	24,4	263,482	23,3
Unskilled workers	98,527	10,6	109,031	11,0	100,740	9,3	133,222	11,8
Domestic workers	61,788	6,6	65,018	6,6	77,037	7,1	78,679	7,0
Agricultural wage earners	105,328	11,3	103,431	10,4	86,554	8,0	68,655	6,1
Total	930,511	100	991,555	100	1,081,055	100	1,129,546	100

Own elaboration on INE datasets. The differences in totals with Table 1 are due to the non-inclusion of cases of employed people without occupational information or who are not identifiable, according to each census.

### 3.1 Company directors and senior public officials

This category includes company directors and general managers, whether employers or salaried employees, across urban and rural sectors. It also comprises senior public officials, including members of the executive, legislative, and judicial branches; directors of state-owned enterprises and public services; and senior officials in territorial governments.

### 3.2 Professionals in specific functions

This group includes university-trained professionals from all fields who are employed in their area of specialization, whether as self-employed or salaried workers.

### 3.3 Small business owners

This category comprises owners who are directly involved in the production process, regardless of whether they employ salaried labor. It includes small entrepreneurs, managers, and retailers.

### 3.4 Technical and similar staff

This group consists exclusively of salaried workers in specialized technical functions. It includes deputy managers and supervisors in private firms, as well as technical and intermediate administrative staff (“cadres”) in the civil service. Teachers at the primary, secondary, tertiary, and university levels, as well as mid-level nurses and auxiliaries, are also included.

### 3.5 Armed forces

This category includes all occupations directly associated with the armed forces, regardless of military rank. It constitutes one of the disaggregated ESO categories.

### 3.6 Administrative and sales employees

This group encompasses salaried office workers, bank clerks, accountants, typists and stenographers, secretaries, surveyors, clerks, promoters, and sales workers in both the public and private sectors. It represents the lower segment of the traditional non-manual sector.

### 3.7 Agricultural producers

This category includes ranchers and farmers, whether employers or self-employed workers. It is one of the disaggregated categories.

### 3.8 Self-employed workers

This group consists of self-employed workers engaged in routine non-manual activities or skilled manual work. It includes non-salaried technicians and teachers, management and sales specialists, and highly specialized non-salaried manual workers. Until the 1975 census, tailors and dressmakers predominated in this category; from 1985 onward, it increasingly included construction technicians, drivers, mechanics, carpenters, kiosk operators, and self-employed market stallholders.

### 3.9 Skilled workers

This category includes salaried skilled manual workers (trades), such as operators and technicians in extractive industries, manufacturing, construction, and metallurgy; plant and machine operators; assemblers; precision mechanics; artisans; craft workers; graphic arts operators; vehicle drivers; and operators of heavy and mobile equipment. It represents the traditional industrial working class and also includes trained personnel in protection and security services (police).

### 3.10 Unskilled workers

This group represents the lower segment of manual labor and includes laborers and journeymen in salaried occupations characterized by high labor turnover, as well as waiters, porters, laundry workers, and janitors.

### 3.11 Domestic workers

This category includes salaried domestic workers employed in private households, such as cooks, cleaners, servants, and nannies. It is highly homogeneous in terms of gender composition and is composed almost entirely of women. It constitutes one of the disaggregated categories.

### 3.12 Agricultural wage earners

This group includes salaried workers engaged in agricultural and forestry activities and represents another disaggregated category.

We also constructed a variable for educational attainment by harmonizing the different ways in which education was measured across the census. For analytical convenience, educational attainment was expressed in years of schooling. We use medians and quantiles by social class for selected cohorts in each census, as discussed in later sections.

## 4 Results of the class structure I: the emergence of the “inverted J”

As indicated above, this study builds on a long tradition of research dating back to the second half of the twentieth century which, through different but largely convergent approaches, has examined changes in Uruguay’s social structure and their relationship to well-being and occupational attainment.

Table 1 and, more specifically, Table 2 show that while the total EAP expanded by approximately 40% over the period, this growth was unevenly distributed across socio-occupational strata (ESOs). Although FG examined the full class structure in their comparative work, particular attention was devoted to the middle and upper strata—hereafter referred to as EMAs—which they regarded as the primary beneficiaries of economic growth and as key to social system stability. Across Latin America, EMAs expanded between the 1950s and the 1970s, albeit at varying rates: in some countries growth was rapid, in others linear, and in others inversely related to initial size. FG adopted Di Tella’s “inverted J” hypothesis to account for these patterns.

While recognizing the role of labor supply dynamics—drawing on Sibley’s (1942) concept of demographic mobility and differential fertility by class—FG emphasized that EMA expansion was fundamentally shaped by labor market structure. This structure both enabled and constrained growth. Consequently, EMA expansion was not concentrated solely in the secondary sector but occurred rapidly within the tertiary sector. FG also stressed that EMAs were not unique in this process; other social classes (here, ESOs) accompanied this sectoral shift.

In our operationalization, EMAs include the following ESOs: Company Directors and Senior Public Officials, Professionals in Specific Functions, Small Business Owners, Technical and Similar Cadres, Armed Forces, and Administrative and Sales Employees. As shown in Table 2, Company Directors and Senior Public Officials exhibited minor fluctuations and ultimately stabilized, tracking the overall growth of the EAP. Small Business Owners displayed oscillatory behavior but maintained their relative size over time. Technical and Similar Cadres declined between 1963 and 1975 but subsequently expanded sharply, growing by approximately 30% by the end of the period. Armed Forces personnel increased substantially between 1975 and 1985, followed by a marked decline, though without returning to their initial levels. Administrative and Sales Employees increased steadily, broadly in line with overall EAP growth.

When EMAs are considered as a whole, their relative weight follows a mild inverted J pattern (29% in 1963, 32% in 1975, 35% in 1985, and 33.8% in 1996), indicating a process of quantitative stabilization. Table 2 does not capture income differentials within EMAs; however, previous work by Errandonea and Bazzi— not developed here—documents substantial income heterogeneity. This is consistent with the expansion of status inconsistencies between income and occupational position in Latin America, a phenomenon highlighted by FG following Heintz, Di Tella, and Germani.

Both rural strata declined over the period. Rural property owners showed initial fluctuations followed by an overall decrease, while rural wage earners declined monotonically. As noted early by Martorelli (1978) and confirmed by subsequent research, deruralization was a persistent process.

Self-employed workers and skilled workers together represented approximately 35% of the EAP throughout the period, but their trajectories diverged. Skilled workers increased by only 15%, whereas self-employed workers increased by nearly 61%, well above the overall growth rate. Although further disaggregation is not possible with the available data, this divergence is clearly linked to the deindustrialization process that intensified following the economic crisis of 1982.

At the lower end of the class structure, urban manual strata—combining unskilled workers and domestic workers—expanded throughout the period. Unskilled workers experienced moderate fluctuations but grew by 35%, while domestic workers increased by 27%.

Overall, these trends reflect the progressive urbanization of both the EAP and the class structure. This process entailed a sustained decline in rural wage labor, though not in rural ownership. It was accompanied by significant and lasting internal migration, which shifted direction after 1975 toward the southeastern region of the country (Bazzi, 2017), contrasting with the historically dominant concentration in Montevideo prior to 1963.

These dynamics underscore the relevance of FG’s “ceiling theory” for EMAs, which is grounded in both labor market demand and state employment capacity. On the demand side, limited opportunities contributed to substantial emigration—approximately 175,000 Uruguayans between 1963 and 1975 (Cabella and Pellegrino, 2005). On the supply side, state employment declined from 23% of total employment in 1963 to 22% in 1975 (despite a 2.2-fold increase in the armed forces), 21.4% in 1985, and 18% in 1996. Although absolute state employment levels remained stable, they did not drive EMA expansion as anticipated by FG. As a result, EMAs reached a ceiling from both directions, consolidating the inverted J pattern by 1996.

According to Bazzi, this process was also unequal in educational terms, as the state increasingly prioritized demand for more highly educated workers—a consequence anticipated by Solari et al. (1967). The following section examines how educational attainment evolved across social classes.

## 5 Results of the class structure II: the role of educational attainment

As shown above, educational expansion intensified throughout the twentieth century. Vallet (2017) and Chauvel (2006) emphasize the central role of education in their analyses of social mobility and social change in France and other European countries, arguing that expansion, universalization, and equalization of opportunities through education substantially increase mobility chances.

In the absence of complete occupational life histories, and given the scope of the present study, we offer a partial analysis. Specifically, we track changes in educational attainment across all ESOs for cohorts of the same age observed in different censuses. This analysis is guided by the hypothesis that educational expansion—reflected in rising enrollment at all levels and increasing years of schooling—was not equally distributed across social classes.

**Figure 3:**
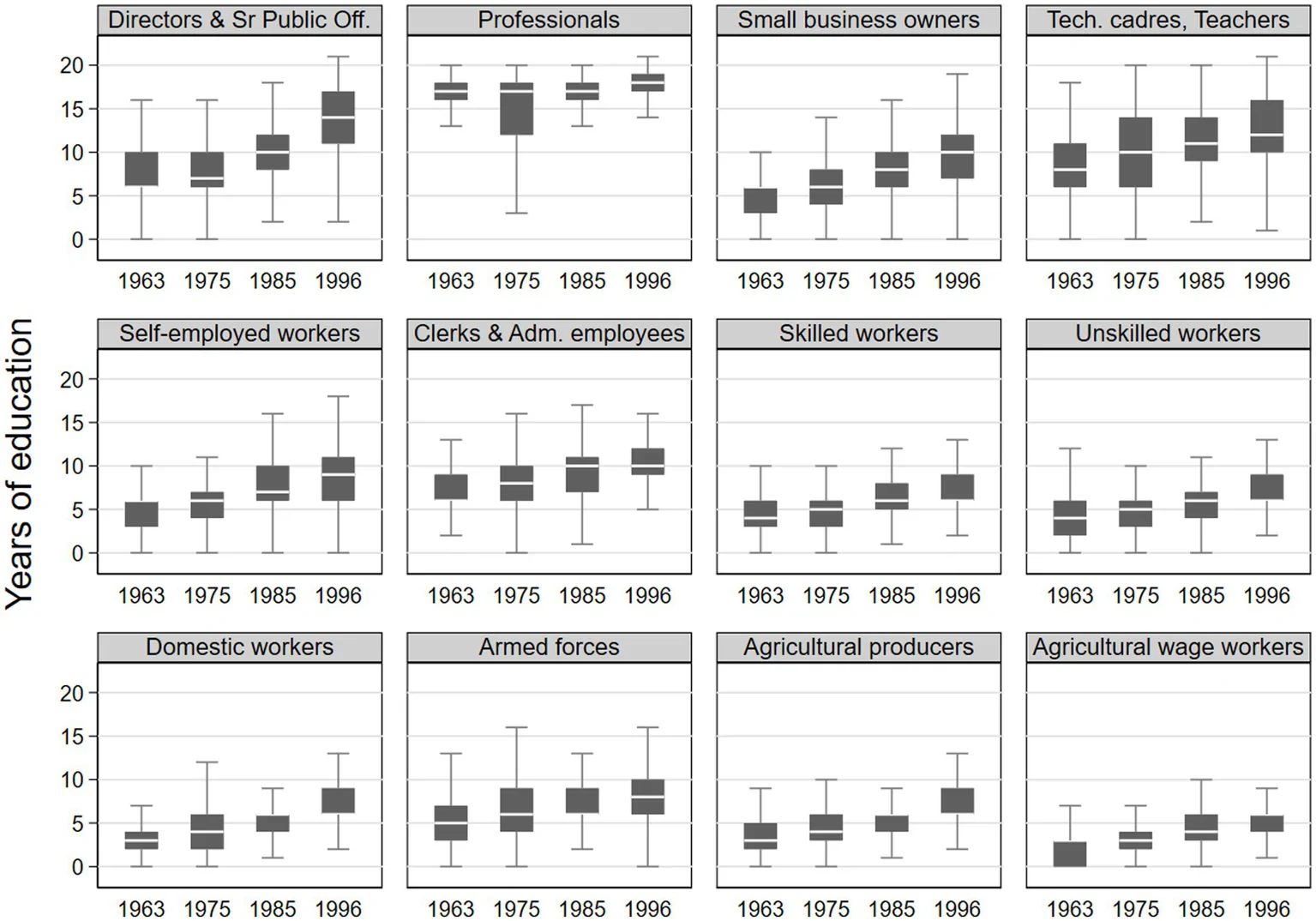
Educational attainment (in years of education) for each ESO according to censuses (box plots: median, 50% interquartile range, maximums and minimums). Own elaboration on INE datasets.

Figure 3 illustrates the impact of educational expansion on ESOs for the full set of EAP census records, using box plots that display both median years of schooling and the central 50% of the distribution for each ESO in each census year^4^.

First, pronounced differences in educational attainment across ESOs are evident. The largest gap separates Professionals in Specific Functions—whose median educational levels consistently exceed 15 years—from Agricultural Wage Earners, whose medians remain below five years. These extremes suggest relatively low internal educational heterogeneity within these ESOs, in contrast to other categories. Notably, the highest-educated ESO nearly tripled in size over the period, while the lowest-educated declined by 35%.

Second, beyond cross-sectional differences, changes over time in median levels and dispersion within each ESO reveal how educational attainment evolved between 1963 and 1996. All ESOs experienced some improvement in educational attainment, although to varying degrees^5^.

Focusing on ESOs within the EMA group, Company Directors, Managers, and Senior Officials—numerically the smallest group—show a striking increase: their median educational attainment rose from six years in 1963 to fourteen years in 1996, surpassing the completion of secondary education and entering tertiary or university levels. After gradual growth between 1963 and 1985, a sharp shift toward professionalization occurred by 1996. Technical and Similar Cadres, representing approximately 10% of the EAP on average, followed a similar trajectory, albeit with less dispersion and more stable career patterns.

Small Business Owners also experienced rising educational attainment, though more modestly, with medians increasing from six to ten years—roughly corresponding to a transition from completed primary to incomplete secondary education. Armed Forces personnel display considerable heterogeneity due to varied training requirements and internal career structures, resulting in limited gains in median educational levels. Administrative and Sales Employees exhibit substantial internal diversity but show gains comparable to Small Business Owners, with medians rising from seven to ten years.

These patterns indicate marked heterogeneity within EMAs and suggest an increasing overlap between property-based and education-based stratification mechanisms that were previously more clearly differentiated. This process—described as the “castling” of economic and human capital—has been noted by Boado (2009), Errandonea (2014), and Bazzi (2017).

Self-employment expanded numerically within the EAP, contradicting the expectation of generalized wage employment predicted by modernization theory. This expansion was accompanied by rising educational attainment, with median years of schooling increasing from six to nine.

A clear divide emerges between manual ESOs (skilled workers, unskilled workers, and domestic workers) and the remaining strata. Together, these groups accounted for approximately 40–42% of the EAP throughout the period yet exhibited much more limited educational advancement. Despite overall educational expansion, their median years of schooling increased only from three in 1963 to six in 1996, and the proportion competing only primary education remained largely unchanged. These ESOs also display the lowest variance in educational attainment, indicating a strong positional component in which additional education yields limited occupational returns.

Finally, agricultural producers benefited only marginally from educational expansion, with median years of schooling increasing from three to seven over the period. Although this represents a doubling of educational attainment, it occurred within a social group that declined in size over time. As noted above, our examination of the effects of education is necessarily limited; nevertheless, it suggests the existence of structural blockages that do not consistently reward educational advancement, alongside pathways that clearly do. As predicted by FG, belonging to the EMA generally favors educational attainment, particularly within those ESOs for which formal education is most directly required.

## 6 Results of the class structure III: the role of women

As shown in Table 1, women’s participation in the EAP increased substantially between 1963 and 1996, rising from 25 to 40%. Consequently, women’s presence expanded across all ESOs^6^. This transformation is especially significant when considered the two major sub-periods analyzed: the final phase of ISI—marked by substantial emigration to Argentina and other countries—and the subsequent phase of economic liberalization and the dismantling of industrial protection.

This process unfolded in the context of pronounced deterioration in real wages (Figure 1) and high inflation. In addition, a factor often overlooked due to limitations in historical data on working hours outside industry was the substantial extension of the working day in public and service-sector employment between 1950 and 1970, from four to six hours and from six to eight hours per day. Given the large size of the service sector (Figure 2), these changes significantly shaped labor demand and conditioned the massive entry of women into the labor market.

**Figure 4:**
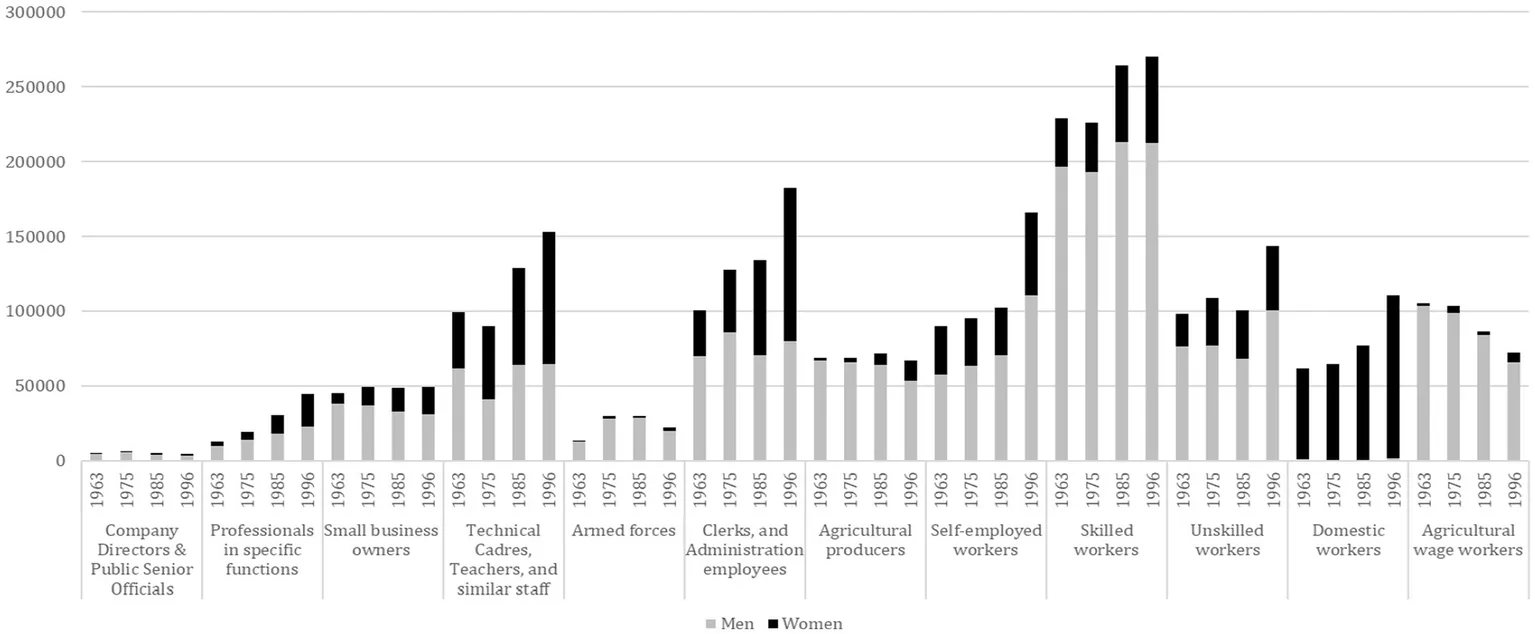
ESOs absolute size intercensuses trends by sex. Source: own elaborations on INE data sets 19663 to 1996.

**Figure 5:**
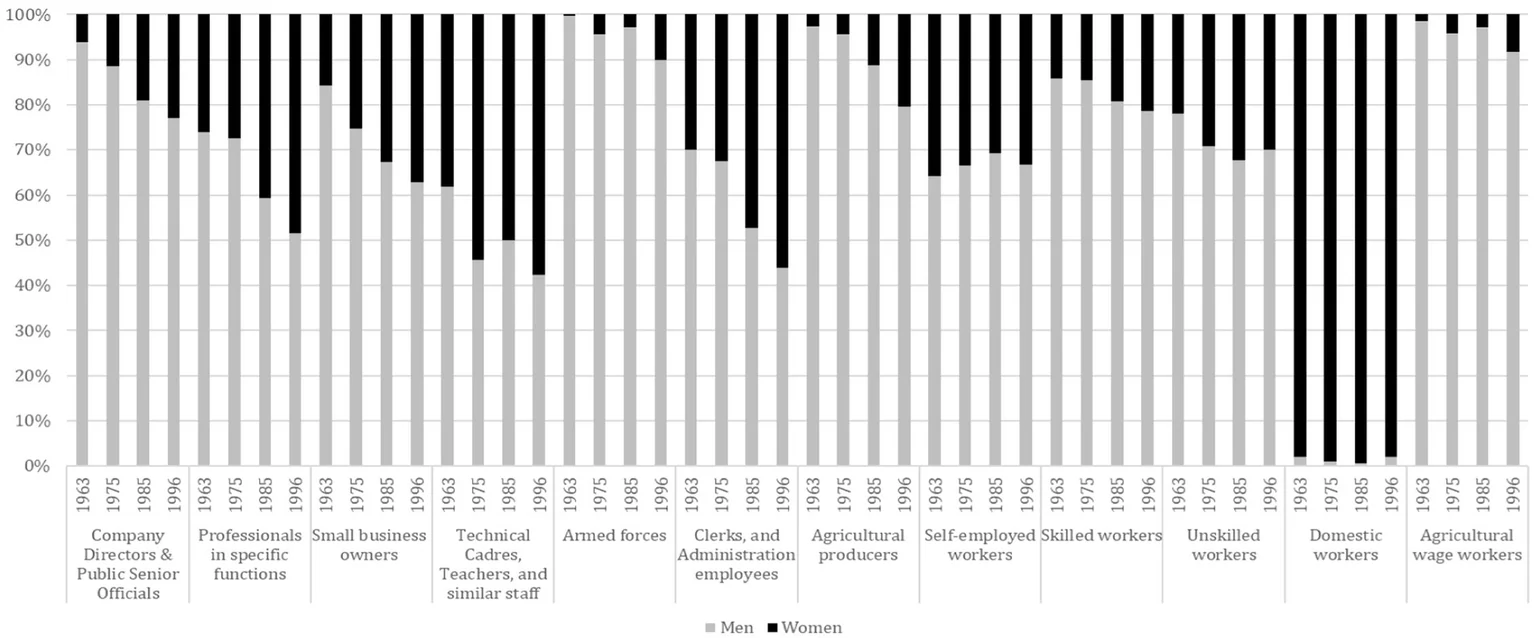
ESOs relative size intercensuses trends by sex. Source: own elaborations on INE data sets 19663 to 1996.

Figures 4, 5 illustrate the absolute and relative evolution of women across ESOs, based on data from Boado and Vanoli (2024). These figures reveal a dual pattern of incorporation. On the one hand, women increasingly entered ESOs located in the upper and middle strata that require higher levels of human capital (the EMA). On the other hand, women also expanded their presence in ESOs concentrated in manual working-class positions with lower educational requirements.

Throughout the 1963–1996 period, domestic employment remained the largest ESO for women, declining in relative but not in absolute terms. At the same time, substantial increases are observed in ESOs such as Salaried Technical and Similar Cadres, Administrative and Sales Employees, and Professionals in Specific Functions. It is therefore reasonable to argue that women’s labor market participation played a key role in sustaining the expansion of several ESOs typically associated with middle-class (EMA) positions and higher educational attainment, particularly toward the end of the second sub-period.

However, women’s advancement within the EMA did not translate into substantial representation among Company Directors and Senior Public Officials, nor among small urban or rural capital owners. This pattern reflects the structural ceiling and the inverted J trajectory predicted by FG for the 1963–1996 period. Figures 4, 5 also show that occupational segregation intensified during the late ISI phase and the early years of economic openness—most clearly in the first three censuses—but began to reverse by the fourth census, driven by the increasing presence of women in professional occupations.

## 7 Conclusion

This article is the outcome of an initially unexpected but ultimately necessary research effort on structural social mobility, aimed at strengthening the analytical foundations of our ongoing work on intergenerational social mobility. This objective justified the emphasis placed on the initial methodological steps: harmonizing census microdata into a single registry, constructing a unified occupational dictionary, and applying a reliable and flexible class scheme based on Torrado (1992) and Boado and Vanoli (2024). The availability of harmonized microdata makes it possible, for the first time, to apply consistent definitions across the entire 1963–1996 period and to measure transformations in the Socio-Occupational Strata (ESO) with a high degree of precision.

Beyond this core objective, the adopted approach allows for further analytical extensions, including the disaggregation of information to assess equality and inequality of opportunity across cohorts and to identify the scope of historical change. In this sense, the framework developed here enables a more precise specification of cohort effects in subsequent analyses of social mobility.

In this study, we conducted a structural–historical analysis based on intertemporal contrasts across ESOs, considering elapsed time, sex, and selected cohorts defined by educational attainment. Our analysis builds on the descriptive contributions of Terra, Errandonea, and Bazzi, while returning to the propositions advanced by Filgueira and Geneletti (FG), whose work offered a dissonant perspective within structural–historical comparison by rejecting a predominantly industrialist theory of stratification. This perspective aligns closely with Uruguay’s twentieth-century trajectory of urbanization, territorial organization, industrial development, and agricultural production.

The main findings can be summarized as follows.

First, in line with the extensive literature on individual intergenerational social mobility, social mobility is induced by macroeconomic transformations that reshape the sectoral composition of the economy and the labor market. The Uruguayan case reinforces FG’s thesis that these transformations were not consistently led by the secondary sector. Agricultural employment declined, the industrial sector failed to absorb the displaced labor force, and the tertiary sector became the primary driver of structural mobility.

Second, these transformations produced not only a distinctive sectoral configuration but also a polarized ESO structure. Middle and upper strata—identified by FG as EMAs—expanded in a dual and uneven manner. One segment consisted of university-trained professionals, technical and intermediate cadres, and senior public and private managers; the other was composed of administrative, routine sales, and service employees. Contrary to expectations derived from modernization theory, self-employment also strengthened. These strata were largely concentrated in finance, business services, and public and private service provision. At the same time, the traditional salaried skilled manual sector in manufacturing weakened markedly (Boado, 2009), being partially replaced by employment in construction and in tertiary-sector services. Overall, the social structure evolved toward two poles, as previously noted by Bazzi: an upper pole dominated by non-manual occupations of varying qualification levels and a lower pole concentrated in low-skilled manual work.

Third, the evidence for the 1963–1996 period confirms FG’s central hypothesis regarding the slowdown in the growth of the middle classes—the “inverted J.” Moreover, the quantitative expansion of ESO categories corresponding to EMAs did not necessarily imply qualitative improvement. Prior research by Terra, Errandonea, FG, and Bazzi had already suggested this outcome, and the present analysis reinforces their conclusions, particularly with respect to income heterogeneity. This finding is consistent with documented transfers from wages to profits in the functional distribution of income during the late ISI phase and the early years of economic liberalization (Marmisolle et al., 2023).

Fourth, the pronounced mismatch between rapid educational expansion and occupational structure during the second half of the twentieth century represents a dual challenge to modernization theory. On the one hand, the productive structure was unable to absorb the growing supply of formally educated workers. This mismatch differs from the classical European experience emphasized in modernization narratives, as noted by Germani (1963) and Solari et al. (1967). It must also be understood within the historical context of the dictatorships of the 1960s and 1970s, which relaxed constraints on certain middle strata while reinforcing pre-existing inequalities, at least until the onset of globalization in the twenty-first century.

On the other hand, our focused analysis of the relationship between class and educational attainment reveals persistent and historically structured differences in schooling across social classes, even when comparing cohorts of the same age across censuses. Educational expansion was used in a stratified manner, contradicting arguments—such as those advanced by Vallet and others for Europe—regarding the expansive and compositional effects of education on class transformation. Although methodological differences must be acknowledged, the Uruguayan case clearly exhibits lower overall educational attainment, high variance in educational outcomes, and enduring inequalities, as documented elsewhere (Boado and Fernández, 2010).

Fifth, the incorporation of women into the labor market constituted a major dimension of structural change for which modernization theory offered limited explanatory tools, beyond the political context of post-war social reform in Western Europe. In Latin America—and in Uruguay in particular—this process followed a polarized trajectory. An initial phase, associated with the end of ISI, was dominated by domestic service, followed by moderately skilled industrial employment and the systematic expansion of public administration. A second and broader phase, linked to economic openness, affected nearly all ESOs. This phase combined significant gains in medium- and high-skilled professional occupations and routine salaried non-manual work—key components of the EMA—with the persistence of low-skilled occupations, especially domestic service, which grew more slowly than the EAP as a whole. Women’s labor market incorporation was highly unequal in income terms, both among women and relative to men, and was predominantly urban, as noted by Bazzi.

For research on individual intergenerational social mobility, the structural–historical framework developed here is particularly valuable. It situates individual trajectories within a long-term perspective on opportunity structures and highlights the persistence of structural cleavages, even when class categories differ from those used in the present analysis.

The overall class structure exhibits considerable inertia, yet this does not preclude meaningful change. The enduring presence of the ESO of Directors, Managers, and Senior Public and Private Officials is accompanied by stable income advantages and rising educational attainment, including the growing participation of women, albeit still below the EAP average (Boado, 2009; Boado and Vanoli, 2024).

Other ESOs also display distinctive trajectories. When skilled, unskilled, and domestic manual workers are considered jointly, they continue to represent approximately 42% of the EAP. These groups benefited unevenly from educational expansion while simultaneously facing economic pressures from policies that reduced labor’s share of GDP and constrained collective bargaining.

Agrarian property owners experienced fluctuating but generally declining trajectories, consistent with broader economic analyses documenting the slowdown of the agricultural sector until the early twenty-first century. Urban property ownership and self-employment reflected small- and medium-scale capitalization strategies but remained vulnerable and exposed to recurrent crises.

As noted throughout the analysis, women’s advancement within the class structure was polarized rather than dissonant with broader structural trends. The persistence of low-skilled female employment, particularly in domestic service, did not disappear but was reshaped by the overall increase in women’s labor force participation. At the same time, substantial gains occurred in administrative employment, technical occupations, senior management, and especially professional roles.

Previous research has shown that women’s educational attainment does not fully converge with that of men (Boado and Fernández, 2010). This pattern, while widespread internationally, requires deeper explanation, as it underpins women’s differentiated progress across ESOs.

Overall, the advance of middle and upper strata in educational attainment challenges the widespread assumption of education as a universal equalizer and casts doubt on strong meritocratic interpretations. As emphasized by FG, Solari, and Germani, the early and persistent mismatch between education and occupations in Latin America warrants close scrutiny. Educational expansion did not automatically universalize middle-class growth; instead, its effects were heterogeneous and class-specific.

Today, the availability of higher-quality and more comprehensive data than those accessible to earlier scholars enables a more rigorous evaluation of these long-standing hypotheses through period- and cohort-based reconstructions, as is standard in contemporary studies of intergenerational social mobility. Future research will build on this reconstructed structural framework to advance that agenda.

## Data Availability

The datasets presented in this study can be found in online repositories. The names of the repository/repositories and accession number(s) can be found at: https://www.gub.uy/instituto-nacional-estadistica/censosphv.

## Footnotes

1 All adjusted by the PPP method to 1990 US dollars. The growth index for all variables takes the year 1913 – the end of the first globalization – as the baseline. The GDP information series is an estimate from various sources (Moxlad, Maddison, BROU, and BCU), not an annual record, and the same is true for population (for the 1908 to 1963 interval).

2 Several currents within sociology have social class as the central variable of analysis, based on the grouping of individuals, or households, into different categories that derive from the structure of production, employment and social power. It is a powerful variable to explain other processes such as political participation, the distribution of welfare, educational qualifications, ways of life, etc.

3 The International Standard Classification of Occupations (ISCO) is a dictionary with definitions of occupations by their actions, with a high level of detail and disaggregation, made to conventionally classify the occupations of countries, created (updated and recommended) by the International Labour Organization, with several versions (1958, 1968, 1988, 2008). The steps of harmonisation of this work are developed in another specific document by Vanoli and Boado (2023, mimeo), where the decision to homogenise all records to the ISCO-88 dictionary (ISCO88) to 2 digits is based on the coding particularities of the revised censuses.

4 To test this hypothesis in a descriptive and bivariate way with a certain degree of control, we made some adjustments, which reduce the scope of our conclusions, but allow progress in the desired direction. We compared the distribution of educational attainment for each social class through the 4 census measurements, but for a single cohort: those who in that census measurement were between 35 and 45 years of age, a period of stability in their working career, without discriminating. This age selection sought to eliminate the effects of the different age structures of the populations for each census, or if observed through the cohorts, the effect of the increase in ages for each generation in successive censuses, due to the irregular periodicity of them. We used the 1920–1929 cohort for the 1963 census, the 1930–1939 cohort for the 1975 census, the 1940–1949 cohort for the 1985 census, and the 1950–1959 cohort for the 1996 census.

5 Except for professional ESO, which presupposes a high level of schooling.

6 Since the beginning of development studies in Latin America, the rate of female participation in the EAP has been considered an indicator of development.
